# Caffeine increases myoglobin expression via the cyclic AMP pathway in L6 myotubes

**DOI:** 10.14814/phy2.14869

**Published:** 2021-05-15

**Authors:** Takumi Yokokawa, Takeshi Hashimoto, Nobumasa Iwanaka

**Affiliations:** ^1^ Faculty of Sport and Health Science Ritsumeikan University Shiga Japan; ^2^ Laboratory of Sports and Exercise Medicine Graduate School of Human and Environmental Studies Kyoto University Kyoto Japan; ^3^ College of Gastronomy Management Ritsumeikan University Shiga Japan; ^4^ Faculty of Health Science Kyoto Koka Women's University Kyoto Japan

**Keywords:** caffeine, cyclic AMP, myoglobin, protein kinase A, skeletal muscle

## Abstract

Myoglobin is an important regulator of muscle and whole‐body metabolism and exercise capacity. Caffeine, an activator of the calcium and cyclic AMP (cAMP)/protein kinase A (PKA) pathway, enhances glucose uptake, fat oxidation, and mitochondrial biogenesis in skeletal muscle cells. However, no study has shown that caffeine increases the endogenous expression of myoglobin in muscle cells. Further, the molecular mechanism underlying the regulation of myoglobin expression remains unclear. Therefore, our aim was to investigate whether caffeine and activators of the calcium signaling and cAMP/PKA pathway increase the expression of myoglobin in L6 myotubes and whether the pathway mediates caffeine‐induced myoglobin expression. Caffeine increased myoglobin expression and activated the cAMP/PKA pathway in L6 muscle cells. Additionally, a cAMP analog significantly increased myoglobin expression, whereas a ryanodine receptor agonist showed no significant effect. Finally, PKA inhibition significantly suppressed caffeine‐induced myoglobin expression in L6 myotubes. These results suggest that caffeine increases myoglobin expression via the cAMP/PKA pathway in skeletal muscle cells.

## Graphical abstract

Myoglobin is an important regulator of muscular and whole‐body metabolism and exercise capacity. However, the detailed molecular pathway regulating the myoglobin expression remains mostly unclear. This study shows that caffeine increases myoglobin expression via the cAMP/PKA pathway in muscle cells.

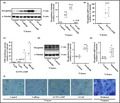

## 1 INTRODUCTION

Myoglobin is widely accepted as a temporary store of oxygen (Kooyman & Ponganis, 1998) and is suggested to release oxygen for mitochondrial consumption and to regulate mitochondrial function (Braganza et al., 2019; Wittenberg & Wittenberg, 1987; Yamada et al., 2015). Myoglobin is also believed to facilitate oxygen diffusion within the cell (Merx et al., 2001); however, reports have been conflicted (Jürgens et al., 2000). In addition to these conventionally known functions, myoglobin acts as a scavenger of nitric oxide (Flögel et al., 2001) and reactive oxygen species (Flögel et al., 2004). Myoglobin knockout mice display impaired endurance capacity and decreased oxygen consumption under resting conditions (Merx et al., 2005). The knockout mice also show a shift to increased glucose and reduced fatty acid utilization in the heart (Flögel et al., 2005). In the heart of the knockout mice, glucose transporter 4 was increased, whereas proteins involved in mitochondrial β‐oxidation were decreased (Flögel et al., 2005). These studies suggest that myoglobin is an important regulator of muscle and systemic metabolism and exercise capacity.

Physiological stimuli regulate protein expression level of myoglobin in cardiac and skeletal muscle. Exercise training increases the expression of myoglobin in skeletal muscle (Lawrie, 1953). In cardiac muscle, hypoxia exposure upregulates the expression of myoglobin (Mammen et al., 2003). However, the detailed molecular pathway regulating myoglobin expression remains unclear. Previous studies reported that myoglobin expression is regulated by a few molecules, such as peroxisome proliferator‐activated receptor γ coactivator‐1 (PGC‐1α; Lin et al., 2002) and Ca^2+^/calmodulin‐dependent protein kinase IV (CaMKIV; Lee et al., 2014); however, there have been a few conflicting reports (Akimoto et al., 2004; Geng et al., 2010). Hence, further studies, including pharmacological investigations, are warranted to uncover the molecular mechanism underlying enhanced myoglobin expression.

Caffeine, a xanthine alkaloid, has been used as a ryanodine receptor agonist for inducing calcium release from intracellular calcium stores (Dettbarn et al., 1994). In skeletal muscle cells and tissue, caffeine activates calcium signaling and subsequently enhances glucose uptake, fat oxidation, and mitochondrial biogenesis (Ojuka, Jones, Han, et al., 2002; Raney & Turcotte, 2008; Wright et al., 2004, 2007). Additionally, caffeine inhibits phosphodiesterase activity, leading to increased cellular cyclic adenosine monophosphate (cAMP) concentration, which is followed by activation of protein kinase A (PKA) and cAMP response element‐binding protein (CREB; Al‐Wadei et al., 2006). The cAMP pathway positively regulates fatty acid oxidation (Gerhart‐Hines et al., 2011) and mitochondrial biogenesis (Yoboue et al., 2012). Thus, caffeine may induce metabolic adaptations in skeletal muscle via the calcium and cAMP pathway. In this context, a previous study had reported that caffeine increased myoglobin 2‐kb promoter activity (Kanatous et al., 2009); therefore, it is likely that caffeine increases myoglobin expression, which may partly contribute to these metabolic adaptations. However, no study has shown that caffeine increases the endogenous gene and protein expression of myoglobin in muscle cells. Further, the molecular mechanism underlying the regulation of myoglobin expression remains unclear.

Based on the aforementioned evidence, we hypothesized that caffeine upregulates myoglobin expression via the cAMP and calcium signaling pathways in myotubes. To test this hypothesis, we investigated whether caffeine and activators of the cAMP and calcium signaling pathways increase the expression of myoglobin in L6 myotubes and whether the pathway mediates caffeine‐induced myoglobin expression.

## 2 METHODS

### 2.1 Cell culture

The rat L6 cell line (American Type Culture Collection) was cultured in 5% CO_2_ atmosphere at 37°C, as described previously (Yokokawa et al., 2015). Myoblasts were grown in Dulbecco's modified Eagle's medium (DMEM; 4.5 g glucose/L; Wako) containing 10% fetal bovine serum (FBS) and 1% penicillin–streptomycin (P/S). The cells were cultured in six‐well plates for western blotting and real‐time polymerase chain reaction (PCR) and in 24‐well plates for the luciferase assay. When myoblasts were confluent, differentiation was induced by switching to differentiation medium containing 2% horse serum and 1% P/S. After 5 days of differentiation, myotubes were stimulated in differentiation medium containing 5 mM caffeine (Nacalai Tesque), 5–500 µM 8‐(Bennett et al., 1996) adenosine 3′,5′‐cyclic monophosphate sodium salt (8‐CPT‐cAMP; Enzo Life Sciences), 0.5 mM 4‐chloro‐*m*‐cresol (4‐CmC; Sigma‐Aldrich), 10 µM forskolin (LKT Laboratories), 10 µM dantrolene (Sigma‐Aldrich), and/or 1 µM KT5720 (Calbiochem). These lipophilic compounds were dissolved in dimethyl sulfoxide (DMSO) and then diluted in the stimulation medium; a corresponding amount of DMSO was used as the vesicle control. The concentration was determined based on previous studies (Ojuka, Jones, Han, et al., 2002; Westerblad et al., 1998) and our pilot studies. The stimulation medium was changed every day. Stimulated myotubes were harvested for luciferase assay, real‐time PCR, or western blotting.

### 2.2 Trypan blue staining assay

To assess cell viability, we performed trypan blue staining. Myotubes were washed with phosphate‐buffered saline and incubated in 0.4% trypan blue solution at room temperature for 5 min. Thereafter, cell viability was visually assessed using a light microscope (i.e., nonviable cells were stained, whereas viable cells were unstained). Cells exposed to methanol, which causes drastic cell death, for 5 min were used as the positive control.

### 2.3 Luciferase assay

To measure cAMP response element (CRE) transcriptional activity, L6 myoblasts were transfected with pGL4.29[*luc2P*/CRE/Hygro] (E847A; Promega) using Lipofectamine 2000 (Thermo Fisher Scientific). Six hours after transfection, the culture medium was changed to DMEM containing 10% FBS. The transfected cells were treated with 5 mM caffeine or 5–500 µM 8‐CPT‐cAMP for 6 h. Then, luciferase assay was performed using a kit (E1910; Promega) according to the manufacturer's instructions.

### 2.4 Quantitative real‐time PCR

Quantitative real‐time PCR was performed as described previously (Yokokawa et al., 2015). Total RNA was extracted using PureLink RNA Mini Kit (Thermo Fisher Scientific) and reverse‐transcribed into cDNA using ReverTra Ace qRNA RT Master Mix with gDNA Remover (Toyobo). Gene expression level was measured using primer sets and Universal ProbeLibrary Probes (Roche Diagnostics), both of which are listed in Table 1. In the pilot study, we validated PCR efficiency using standard curves. Target gene expression levels were normalized to the expression level of glyceraldehyde‐3‐phosphate dehydrogenase (GAPDH).

**TABLE 1:** Primer and probe sets used for real‐time PCR

mRNA	Sequence (5ʹ−3ʹ)		Probe number
Myoglobin	Forward	agggacaacatgctgctga	110
	Reverse	tcttcaggacttggatgatgac	
GAPDH	Forward	catcgtggaagggctcat	158
	Reverse	cgccacagctttccagag	

### 2.5 Western blotting

Western blotting was performed as described previously (Yokokawa et al., 2018). Briefly, stimulated cells were lysed in radioimmunoprecipitation buffer supplemented with protease and phosphatase inhibitor. Lysates in equal volume were subjected to sodium dodecyl sulfate‐polyacrylamide gel electrophoresis, and the separated proteins were transferred to polyvinylidene difluoride membranes. Target proteins were probed with primary antibodies against myoglobin (sc‐25607; Santa Cruz Biotechnology), phospho‐CREB Ser^133^ (9198; Cell Signaling Technology), phospho‐5′‐adenosine monophosphate‐activated protein kinase (AMPK)α Thr^172^ (2535; Cell Signaling Technology), and α‐tubulin (G9545; Sigma‐Aldrich) and appropriate secondary antibodies. Digital images were obtained using Immobilon Forte Western HRP Substrate (Millipore) and ImageQuant LAS 4000 (GE Healthcare). Immunoblotting images were analyzed using Fiji software (NIH). The signal intensities of target proteins on the immunoblots were normalized to those of α‐tubulin.

### 2.6 Statistical analysis

All statistical analyses were performed using R software (Ihaka & Gentleman, 1996). Two‐tailed Welch's *t* tests were used for comparisons between two groups. Parametric (for normally distributed data) or nonparametric (for skewed data) multiple comparison tests were performed using Benjamini–Hochberg method. An interaction of two factors was analyzed by two‐way ANOVA. All violin plots were produced using the ggplot2 package in R. The statistical significance level was set to *p* < 0.05.

## 3 RESULTS

### 3.1 Caffeine upregulates myoglobin expression in L6 myotubes

**FIGURE 1:**
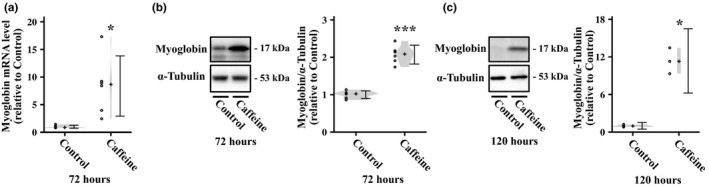
Effect of caffeine on the gene and protein expression of myoglobin in L6 myotubes. (a) L6 myotubes were incubated with 5 mM caffeine for 72 h, and then, myoglobin mRNA expression was measured by real‐time PCR (*n* = 6 per group). (b, c) L6 myotubes were stimulated with caffeine for (b) 72 or (c) 120 h, and myoglobin protein expression was measured by western blotting (*n* = 3–6 per group). Representative immunoblots and quantification of protein expression levels of myoglobin are shown. Distributions of values are depicted as violin plots; dot plots represent individual data points; cross signs depict the median in each group; error bars show 95% confidence intervals. *p < 0.05, ***p < 0.001 versus Control. Statistical significance was assessed by Welch's *t* tests.

We evaluated the effect of caffeine treatment on myoglobin expression in myotubes. Treatment with 5 mM caffeine for 72 h significantly increased the gene expression of myoglobin in L6 myotubes (Figure 1a). Additionally, the protein level of myoglobin was significantly upregulated by caffeine treatment for 72 (Figure 1b) and 120 h (Figure 1c).

### 3.2 Caffeine activates CREB in L6 myotubes

**FIGURE 2:**
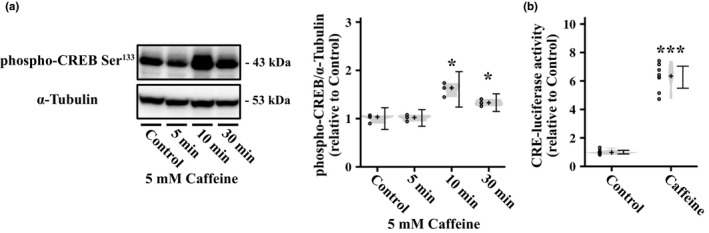
Effect of caffeine on cyclic AMP (cAMP) pathway and CRE promoter activity in L6 myoblasts and myotubes. (a) L6 myotubes were stimulated with 5 mM caffeine for 0, 5, 10, or 30 min, and then, phospho‐CREB Ser^133^ level was measured by western blotting (*n* = 3 per group). Representative immunoblots and quantification of phosphorylation levels of CREB Ser^133^ are shown. (b) L6 myoblasts were incubated with 5 mM caffeine for 6 h, and then, CRE‐luciferase activity was measured by luciferase assay (*n* = 8 per group). Distributions of values are depicted as violin plots; dot plots represent individual data points; cross signs depict the median in each group; error bars show 95% confidence interval. * *p* < 0.05, *** *p* < 0.001 versus Control. Statistical significance was assessed by a multiple comparison test with (a) the Benjamini–Hochberg method or (b) a Welch's *t* test.

To investigate whether caffeine activates PKA in myotubes, we assessed the effect of caffeine on the phosphorylation level of CREB Ser^133^, downstream of PKA (Gonzalez & Montminy, 1989), in L6 myotubes. We found that 5 mM caffeine for 10 and 30 min significantly increased the phosphorylation level of CREB Ser^133^ (Figure 2a). Furthermore, we assessed the effect of caffeine on CRE transcriptional activity in L6 myotubes using luciferase assay. Treatment with caffeine significantly upregulated CRE‐mediated transcriptional activity in L6 myotubes (Figure 2b), consistent with a previous study using primary cultured neurons (Connolly & Kingsbury, 2010). These results indicate that caffeine activates the PKA/CREB pathway in myotubes.

### 3.3 Activation of the cAMP pathway increases myoglobin expression in L6 myotubes

**FIGURE 3:**
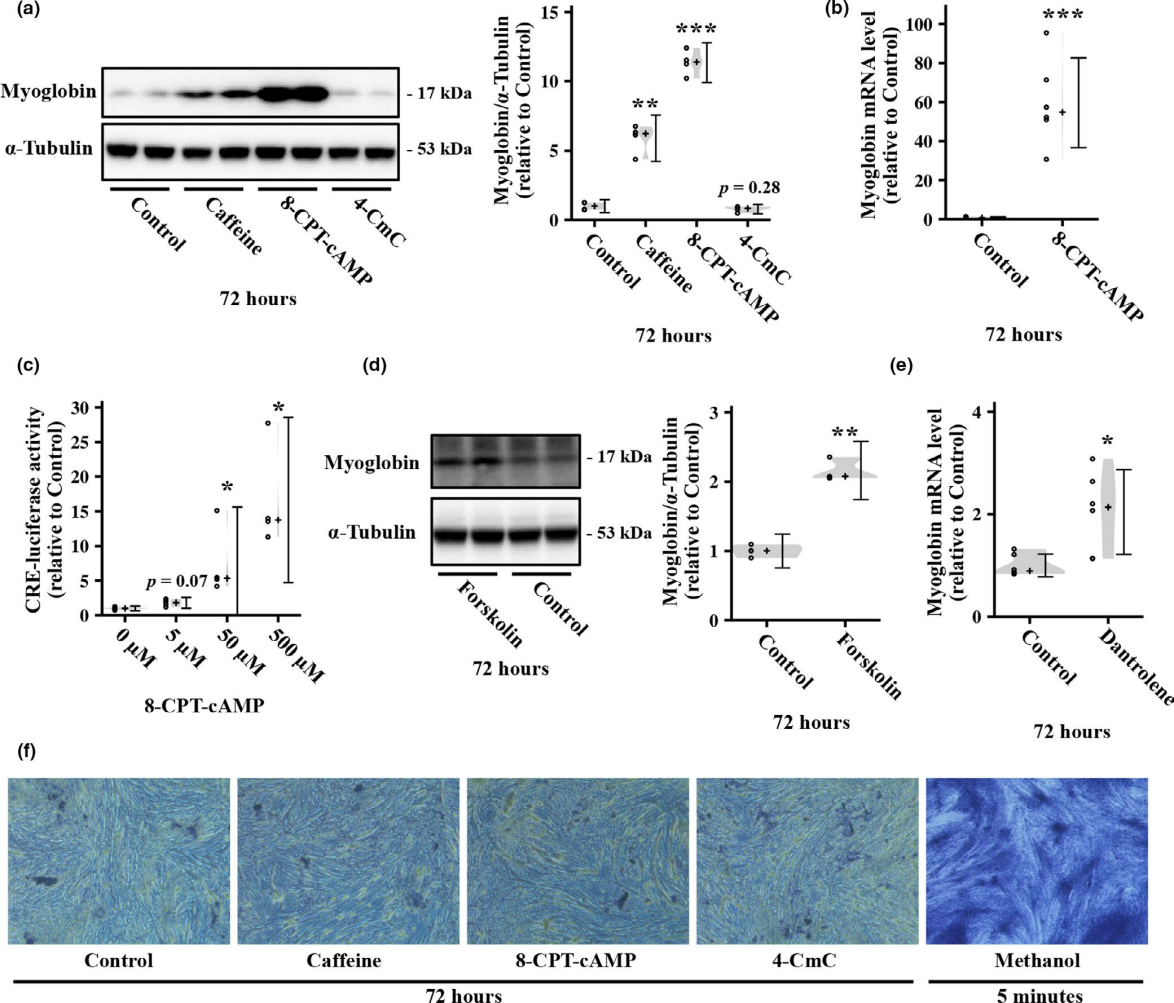
Effect of cyclic AMP (cAMP) and calcium signal activation on myoglobin expression in L6 myotubes. (a) L6 myotubes were stimulated with 5 mM caffeine, 0.25 mM 8‐CPT‐cAMP, or 0.5 mM 4‐CmC for 72 h, and then, myoglobin protein level was measured by western blotting (*n* = 4 per group). Representative immunoblots and quantification of protein expression levels of myoglobin are shown. (b) L6 myotubes were stimulated with 8‐CPT‐cAMP for 72 h, and then, myoglobin mRNA level was measured by real‐time PCR (*n* = 6 per group). (c) L6 myoblasts were incubated with 0, 5, 50, or 500 µM 8‐CPT‐cAMP for 6 h, and CRE‐luciferase activity was then measured by luciferase assay (*n* = 3 per group). (d) L6 myotubes were stimulated with 10 µM forskolin for 72 h, and then, myoglobin protein level was measured by western blotting (*n* = 3 per group). Representative immunoblots and quantification of protein expression levels of myoglobin are shown. (e) L6 myotubes were stimulated with 10 µM dantrolene for 72 h, and the myoglobin mRNA level was then measured by real‐time PCR (*n* = 6 per group). (f) After being treated for 72 h with 5 mM caffeine, 0.25 mM 8‐CPT‐cAMP, or 0.5 mM 4‐CmC, the myotubes were stained with trypan blue. Methanol treatment for 5 min, which induces considerable cell death, was used for the positive control. Distributions of values are depicted as violin plots; dot plots represent individual data points; cross signs depict the median in each group; error bars show 95% confidence interval. * *p* < 0.05, ** *p* < 0.01, *** *p* < 0.001 versus Control. Statistical significance was assessed by (a) parametric or (c) nonparametric multiple comparison tests with the Benjamini–Hochberg method or (b, d, and e) Welch's *t* test.

To investigate whether the cAMP and calcium signaling pathway are involved in regulation of myoglobin expression in myotubes, we treated L6 myotubes with 8‐CPT‐cAMP, a cell‐permeable cAMP analog, and 4‐CmC, an activator of ryanodine receptors, for 72 h. As with caffeine, 250 µM 8‐CPT‐cAMP treatment increased the protein expression of myoglobin in L6 myotubes (Figure 3a). Similarly, 8‐CPT‐cAMP upregulated myoglobin mRNA (Figure 3b) and CRE‐mediated transcriptional activity (Figure 3c). Further, to assess the role of cAMP pathway in myoglobin induction, we also investigated whether myoglobin expression is increased by another cAMP pathway activator forskolin, which activates adenylate cyclase and increases the intracellular cAMP concentration (Ammon & Müller, 1985). Consequently, we found that treatment with forskolin for 72 h increased the protein level of myoglobin in L6 myotubes (Figure 3d). These results suggest that the cAMP pathway positively regulates myoglobin expression in myotubes. In contrast, 0.5 mM 4‐CmC did not induce a significant change in myoglobin expression (Figure 3a), although this concentration is reportedly sufficient to increase the cytosolic calcium concentration in skeletal muscle cells (Freymond et al., 2002; Westerblad et al., 1998). Moreover, we unexpectedly observed that treatment with dantrolene, a ryanodine receptor antagonist, for 72 h increased myoglobin gene expression (Figure 3e). To verify the effects of caffeine, 8‐CPT‐cAMP, and 4‐CmC on cell viability, we performed a trypan blue staining assay and observed no drastic effects of these stimuli; methanol exposure expectedly induced salient cell death (Figure 3f).

### 3.4 Inhibition of PKA suppresses caffeine‐induced myoglobin expression in L6 myotubes

**FIGURE 4:**
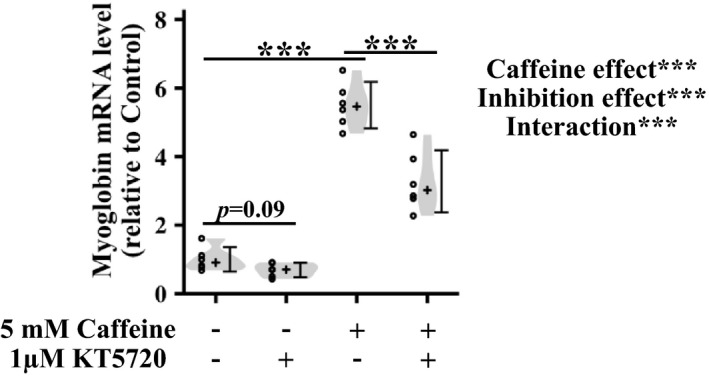
Effect of protein kinase A inhibition on caffeine‐induced myoglobin expression in L6 myotubes. The cells were incubated with or without 5 mM caffeine and 1 µM KT5720 for 72 h before measuring the myoglobin gene expression level by real‐time PCR. Distributions of values are depicted as violin plots; dot plots represent individual data points; cross signs depict the median in each group; error bars show 95% confidence intervals. *n* = 6 per group. *** *p* < 0.001. Data were analyzed by two‐way ANOVA and a multiple comparison test adjusted with the Benjamini–Hochberg method.

We evaluated whether the cAMP/PKA pathway mediates caffeine‐induced myoglobin induction in myotubes. Caffeine treatment for 72 h increased myoglobin mRNA expression in L6 myotubes (Figure 4). To inhibit PKA activity, we used KT5720, a competitive antagonist of ATP at its binding site on the PKA catalytic subunit (Murray et al., 2008). Treatment with KT5720 without caffeine only showed a trend of decrease in myoglobin mRNA expression compared with nontreated control (Figure 4). In contrast, KT5720 significantly suppressed caffeine‐induced increase in myoglobin mRNA (Figure 4).

## 4 DISCUSSION

In this study, we found that caffeine increased myoglobin expression and CRE‐mediated transcriptional activity in L6 muscle cells. We also observed that cAMP analog, but not 4‐CmC, upregulated myoglobin expression. Finally, PKA inhibition suppressed caffeine‐induced myoglobin induction in L6 myotubes. This is the first study showing that caffeine increases myoglobin expression in L6 myotubes, which is at least partly mediated by the cAMP/PKA pathway.

A previous study had reported that caffeine enhances exogenous myoglobin promoter activity in C2C12 muscle cells (Kanatous et al., 2009). However, it was unclear whether caffeine increases the endogenous gene and protein expression of myoglobin in muscle cells. In this study, we found that caffeine upregulated the gene and protein expression of myoglobin in L6 myotubes. Previous studies using myoglobin knockout mice showed that myoglobin is required for the integrity of exercise capacity and muscle and whole‐body metabolism (Merx et al., 2005). Additionally, myoglobin is suggested to be involved in the regulation of nitric oxide (Flögel et al., 2001) and reactive oxygen species (Flögel et al., 2004). Furthermore, exercise training upregulates myoglobin expression in skeletal muscle (Lawrie, 1953). Thus, our results suggest that caffeine increases myoglobin protein expression in skeletal muscle, likely contributing to improved exercise capacity and metabolism. On the contrary, although we used 5 mM caffeine according to previous studies on L6 myotubes (McConell et al., 2010; Ojuka et al., 2003; Ojuka, Jones, Han, et al., 2002; Ojuka, Jones, Nolte, et al., 2002), this concentration is supraphysiological (reviewed in Tallis et al., 2015). Therefore, it is unclear whether caffeine increases myoglobin expression in vivo, and there is requirement for further in vivo investigation to uncover the physiological significance of our in vitro study.

Caffeine has been widely used to elicit exercise‐like intracellular calcium change in skeletal muscle cells (Ojuka et al., 2003; Wright et al., 2007). Pharmacologically, caffeine acts as an agonist of ryanodine receptors and activates calcium signaling (Dettbarn et al., 1994). A previous study reported that myoglobin promoter activity is positively regulated by the calcineurin/nuclear factor of activated T‐cells (NFAT) pathway (Kanatous et al., 2009), which is activated by enhanced cytosolic calcium concentration via ryanodine receptors. Therefore, caffeine‐induced myoglobin induction is suggested to be mediated by calcium signaling and subsequently the calcineurin/NFAT pathway (Kanatous et al., 2009). Furthermore, dantrolene diminished caffeine‐induced PGC‐1α expression and mitochondrial biogenesis in L6 myotubes (Ojuka et al., 2003); hence, it is plausible that caffeine‐induced calcium signaling also regulates myoglobin expression. However, in this study, 4‐CmC, an activator of ryanodine receptors, did not significantly change the protein expression of myoglobin in L6 myotubes. Moreover, we unexpectedly found that treatment with dantrolene alone increased myoglobin gene expression. Therefore, our results did not support the hypothesis that calcium signaling activated via ryanodine receptors mediates caffeine‐induced myoglobin induction. This discrepancy between previous and our studies may also be explained by the difference in assay methods (exogenous promoter activity vs. endogenous gene and protein expression; Kanatous et al., 2009). Additionally, the expression of ryanodine receptors is specifically induced in differentiated L6 myotubes (Bennett et al., 1996), suggesting that caffeine‐ and 4‐CmC‐induced calcium signaling activation is dependent on the differentiation state under experimental conditions. Interestingly, long‐term treatment with a ryanodine receptor agonist or antagonist may elicit unidentified compensatory adaptation. Hence, further studies using isolated skeletal muscles and in vivo models may reveal the contribution of calcium signaling in caffeine‐induced myoglobin expression.

Caffeine inhibits phosphodiesterase activity leading to increased cellular cAMP concentration (Al‐Wadei et al., 2006). However, the relationship between the cAMP/PKA pathway and myoglobin expression was unclear. In this study, a cAMP analog and other PKA activators upregulate the protein expression of myoglobin in L6 myotubes. In this context, we found that caffeine increases the phosphorylation of CREB Ser^133^ and CRE‐dependent transcriptional activity, consistent with previous studies (Connolly & Kingsbury, 2010; Hughes et al., 2017). Additionally, PKA inhibition using KT5720 suppressed an increase in myoglobin expression by caffeine treatment in L6 myotubes. A limitation to this study was that we measured CRE‐dependent transcriptional activity using myoblasts, not myotubes, due to the low transfection efficiency for myotubes under our experimental conditions. Nevertheless, taken together, our results suggest that caffeine‐induced myoglobin induction is mediated by the cAMP/PKA pathway.

The molecular mechanism between the cAMP/PKA pathway and myoglobin expression remains unclear. Previous studies have reported that muscle‐specific PGC‐1α (Lin et al., 2002) and CaMKIV (Lee et al., 2014) transgenic mice show enhanced myoglobin expression in skeletal muscle. As with PKA, CaMKIV is upstream of CREB, and subsequently, its activation induces CRE‐dependent transcription (Sun et al., 1994). PGC‐1α promoter has a full palindromic consensus CRE site, which is essential for PGC‐1α transcription induced by PKA and CaMKII activation (Handschin et al., 2003; Herzig et al., 2001). Therefore, the cAMP/PKA pathway might positively regulate CREB‐dependent PGC‐1α transcription and then enhance myoglobin expression. However, PGC‐1α (Geng et al., 2010) and CaMKIV (Akimoto et al., 2004) knockout mice fail to show decreased myoglobin expression in skeletal muscle, suggesting that the contribution of both genes in myoglobin expression remains controversial. Since myoglobin, PGC‐1α, and CaMKIV are crucial factors for integral muscle function, undefined compensatory responses to maintain muscle function may be responsible for the inconsistency between gain and loss of function studies. Hence, further investigation is required to uncover the molecular mechanism linking the cAMP/PKA pathway and myoglobin expression.

In addition to the PKA and calcium signaling pathways, previous studies have reported that acute caffeine treatment activates the AMPK pathway in incubated skeletal muscle and in vivo (Egawa et al., 2009, 2011). Since AMPK is an upstream molecule of PGC‐1α activation and induction (Jäger et al., 2007; Jørgensen et al., 2005; Terada et al., 2002), it is plausible that the caffeine‐induced AMPK/PGC‐1α axis positively regulates myoglobin expression. However, in L6 myotubes, caffeine treatment for 1 h has been reported to fail to activate AMPK, suggesting that caffeine‐induced mitochondrial biogenesis is independent of AMPK (McConell et al., 2010). We observed that caffeine treatment for 5 and 10 min increased the phosphorylation of AMPK Thr^172^; however, this increase was reversed after 30 min of treatment (Figure S1). These results suggest that caffeine‐induced AMPK activation in L6 myotubes is transient compared to that in rat skeletal muscle (Egawa et al., 2009). Furthermore, the expression of AMPKα isoforms in L6 myotubes differs from that in skeletal muscle tissue (Deshmukh et al., 2010). These results indicate that the use of L6 myotubes is unsuitable for investigating whether AMPK mediates caffeine‐induced myoglobin induction. Therefore, to rigorously explore the relationship between AMPK and caffeine‐induced myoglobin induction, in vivo studies using AMPK‐knockout or dominant negative mice are required.

In conclusion, this study demonstrated that caffeine treatment activated the PKA pathway and myoglobin expression in L6 myotubes. Additionally, activation of the PKA pathway increased myoglobin expression, and PKA inhibition suppressed caffeine‐induced myoglobin expression. These results suggest that caffeine increases myoglobin expression via the cAMP/PKA pathway in muscle cells.

## CONFLICT OF INTEREST

No conflicts of interest, financial or otherwise, are declared by the authors.

## AUTHOR CONTRIBUTION

Takumi Yokokawa and Nobumasa Iwanaka conceptualized the study. Takumi Yokokawa conducted the formal analysis. Takumi Yokokawa and Nobumasa Iwanaka conducted the investigation. Takumi Yokokawa wrote the original draft preparation. Nobumasa Iwanaka and Takeshi Hashimoto reviewed and edited the manuscript. Takeshi Hashimoto supervised the study. Nobumasa Iwanaka and Takeshi Hashimoto are responsible for funding acquisition. All authors have read and agreed to the published version of the manuscript.

## Supporting information



Fig S1

Fig S1Legend

## Data Availability

Data associated with this study are available from the corresponding author upon reasonable request.
