# Supplementary figures and images for: Caffeine increases myoglobin expression via the cyclic AMP pathway in L6 myotubes
Source: Physiol Rep. 2021 May 15;9(9):e14869. doi: 10.14814/phy2.14869 (PMC8123560; doi:10.14814/phy2.14869)

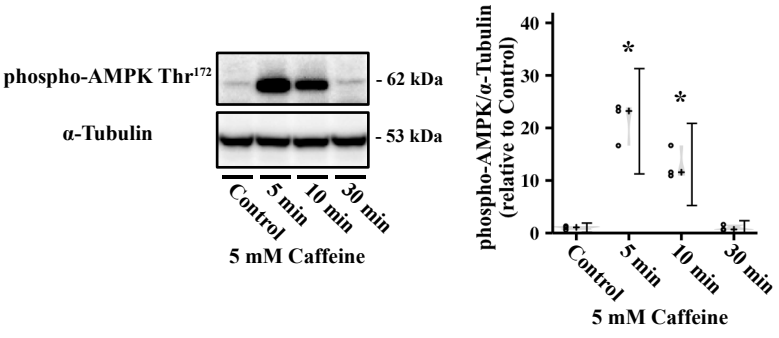

Supplement: Supplementary file 1 — Fig S1 [file PHY2-9-e14869-s001.pdf]
