# Supplementary material for: Caffeine increases myoglobin expression via the cyclic AMP pathway in L6 myotubes
Source: Physiol Rep. 2021 May 15;9(9):e14869. doi: 10.14814/phy2.14869 (PMC8123560; doi:10.14814/phy2.14869)
Supplement: Supplementary file 2 — Fig S1Legend [file PHY2-9-e14869-s002.docx]

Figure S1. Effect of caffeine on AMPK pathway in L6 myotubes. L6 myotubes were stimulated with 5 mM caffeine for 0 (Control), 5, 10, or 30 min (min); thereafter, phospho-AMPKα Thr^172^ level was analyzed through western blotting (*n* = 3 per group). Representative immunoblots and quantification of phosphorylation levels of AMPKα Thr^172^ are shown. Violin plots represent distributions of values; dot plots represent individual data points; cross signs represent the median in each group; error bars show 95% confidence interval. ^*^*P*< 0.05 vs. Control. Statistical significance was assessed by a multiple comparison test using the Benjamini–Hochberg method.
